# Application of Potentiometric and Electrophoretic Measurements to Evaluate the Reversibility of Adsorption of Divalent Ions from a Solution on Titanium Dioxide

**DOI:** 10.3390/molecules29030555

**Published:** 2024-01-23

**Authors:** Wojciech Piasecki, Karolina Lament

**Affiliations:** Department of Physical Education and Health, Józef Piłsudski University of Physical Education in Warsaw, Akademicka 2, 21-500 Biała Podlaska, Poland; karolina.lament@awf.edu.pl

**Keywords:** reversibility, titration, ion adsorption, titanium dioxide, ion-selective electrode, electrokinetic potential

## Abstract

The adsorption of divalent ions on metal oxides is controlled by the pH of a solution. It is commonly assumed that this is a reversible process for pH changes. However, there are reports that the sorption of ions on oxides may not be reversible. To verify this, we used potentiometric titration, ion-selective electrodes (ISEs), and electrokinetic measurements to examine the reversibility of the adsorption of hydrogen ions and three metal ions (Ca^2+^, Cu^2+^, and Fe^2+^) on TiO_2_. The ferrous ion was used as a reference because its adsorption is entirely irreversible. The surface charge determined by potentiometric titration and the adsorption edges measured using ISE indicate that the adsorption of copper ions is reversible with changes in pH. In the case of calcium ions, the results suggest a certain degree of irreversibility. There are apparent differences in the electrokinetic potential data obtained during titration with base and acid, which suggests that the adsorption is irreversible. We have explained this contradiction by considering the complex and dynamic nature of electrophoretic mobility. In our opinion, potentiometric titration may be the simplest and most reliable method for assessing the reversibility of multivalent ion adsorption.

## 1. Introduction

Metal oxide particles in contact with an aqueous solution form the most crucial interface in the environment [1]. The hydroxyl groups on the surface of the oxides immersed in water are active surface sites interacting with ions in the solution. The electric charge of the surface covered by hydroxyl groups (SOH) depends on the pH of the solution. Therefore, pH controls the adsorption of ions on oxides. Electrolytes that interact with the oxide surface only through non-specific electrostatic interactions are called inert electrolytes. These are monovalent salts such as NaCl or $KNO_{3}$ . Divalent ions (e.g, $Ca^{2+}$ , $Pb^{2+}$ , $SO_{4}^{2-}$ ), in addition to electrostatic interactions, can interact with hydroxyl groups on the oxide surface by forming coordination linkages and hydrogen bonds [2]. These stronger and more complex interactions cause such ions to be closer to the oxide surface than monovalent ions. Hence, we have inner-sphere surface complexes (for multivalent ions) and outer-sphere surface complexes (for monovalent ions) [2]. For many years, surface complexation has been a critical concept in understanding oxide reactivity [3,4,5].

Traditionally, depending on the mechanism of interaction of an adsorbent with ions, the adsorption processes can be divided into physisorption, chemisorption, and ion exchange [6]. Since physisorption occurs due to weak intermolecular forces (mainly electrostatic), it is considered a reversible process. On the other hand, chemisorption is based on strong chemical interactions in which atomic orbitals are engaged. Therefore, forming coordination compounds, surface redox reactions, and precipitation can be regarded as chemisorption. The above division is not completely precise but is frequently used.

In order to monitor the process of ion adsorption on oxides, we must measure the pH of the solution and ion concentrations. Additionally, valuable information about changes in electric charge on oxide particles is provided by measuring the electrokinetic potential (ζ) [7]. From precise measurements of pH changes and knowing the exact amounts of acid and base introduced into the system, we can calculate the surface charge generated by hydrogen and hydroxyl ions (i.e., the proton surface charge— $\sigma_{H}$ ) [8,9]. Measuring the loss of divalent ion concentration in the solution as a function of pH allows for determining their adsorption in the form of the so-called adsorption edge [10].

When studying the adsorption process, various spectrophotometric techniques are the most frequently used methods for measuring the concentration of ions in a solution [10]. In this case, measurements using ion-selective electrodes (ISEs) have rarely been used because these electrodes may be sensitive to other ions in the solution and work properly only within a narrow pH range. Recently, we successfully used Ca-ISE to study the adsorption mechanism of calcium ions on metal oxides [11].

Surprisingly, it is tough to find papers in the literature examining the adsorption and subsequent desorption of divalent ions on oxides as a function of pH [12,13,14,15]. The conclusions drawn from these few works are that the adsorption of divalent ions is either irreversible or that the kinetics of their desorption are much slower than the kinetics of adsorption. There are also few publications on the reversibility of the titration of oxide suspensions (forward and reverse titration) [16,17,18]. It seems that the silent assumption was made that the adsorption of divalent ions is a fully reversible process. At the same time, it is believed that these types of ions interact strongly with the oxide surface in a complex manner [2]. So, only their adsorption is tested by gradually increasing the pH, but their desorption is not checked by decreasing the pH.

We verified the above assumptions by examining the adsorption and desorption of divalent ions caused by forward and reverse titration. We chose $TiO_{2}$ as the model oxide [19,20]. It is produced in large quantities by industry and used as a white pigment in paints, a sunscreen in cosmetics, and a photocatalyst [21]. Therefore, large amounts of $TiO_{2}$ nanoparticles end up in the aquatic environment, where they are an important adsorbent of metal ions [22,23,24,25]. Titanium dioxide is a durable and water-insoluble oxide that is stable over a wide pH range, unlike oxides such as $SiO_{2}$ or $Al_{2}O_{3}$ [26].

In our experiments, we examined the adsorption of three ions, $Ca^{2+}$ , $Cu^{2+},$ and $Fe^{2+},$ on titanium dioxide. The first of them is considered to be an ion that interacts relatively weakly with the oxide surface [27]. The iron cation is regarded as a species that interacts strongly during adsorption and adsorbs irreversibly [28]. The concentration of calcium and copper ions as a function of pH was monitored using ion-selective electrodes [29]. The concentration of iron ions in the solution was measured using spectrophotometry. In our experiment, the adsorption of iron ions was a reference, showing the course of entirely irreversible adsorption.

In the presence of divalent ions, we also measured the proton surface charge and electrokinetic potential because these two quantities are the most frequently determined for oxide dispersion in water and provide significant insight into the structure of the electric double layer and how it is formed at the oxide/electrolyte interface.

## 2. Results

A potentiometric acid–base titration is the primary method to determine the surface charge of oxide nanoparticles in a solution. However, using this experimental technique involves many technical difficulties that need to be solved [8,9]. It is worth mentioning some of them.

The sensor used in these measurements is a combined glass electrode. The insensitivity of the electrode potential to the interaction of its surface with charged oxide particles is essential during the titration of an oxide suspension. Converting the measured electrode potential into a pH requires accurate calibration using pH buffers over a wide pH range. The determined pH corresponds to the activity of hydrogen ions in the solution, which must be converted into their concentration. To do this, we need to know the ionic strength of the solution. However, the higher the electrolyte concentration, the more problematic the calculation of the ionic strength becomes. On the other hand, we must remember that we add some amounts of acid or base to the solution during titration, which produces an additional quantity of electrolyte in the neutralization reaction. Simultaneously maintaining a stable ionic strength and its low value, necessary for the precise calculation of the activity coefficients of hydrogen and hydroxide ions, requires operation in a narrow range of electrolyte concentrations. In our surface charge and adsorption measurements, the electrolyte concentration was 0.1 M. In the electrokinetic measurements, it was equal to 0.01 M. Applying a lower ionic strength value while measuring the zeta potential causes a weaker screening of charged particles and gives higher and more reproducible values of the electrokinetic potential [30].

The oxide dispersion may not be stable during titration. Some oxides dissolve quickly at high or low pH values. For example, silica dissolves in an alkaline solution, while aluminum oxide dissolves in an acidic one [9]. Using such an oxide in a titration in which we examine the reversibility of the ion adsorption process with a change in pH will result in more visible hysteresis in the data [9,31]. In the traditional interpretation of the adsorption process, this should mean that the adsorption is partially irreversible. Therefore, selecting an appropriate pH range for the titration of an oxide over the entire measurement range is essential. Titanium dioxide is a stable and insoluble material and was therefore chosen by us as the model oxide [32].

Yet another issue is the agglomeration of oxide nanoparticles, which may occur in dispersion when their surface charge changes. This may be particularly important near the point of zero charge (pzc). This process leads to a reduction in the oxide/solution phase boundary and a slowdown in the ion adsorption and desorption processes [8].

Technically, the surface charge of an oxide is expressed in $C/m^{2}$ , so in addition to knowing the charge, we also need to know the specific surface area of the oxide. This quantity is most often measured using nitrogen adsorption on a “dry” oxide (BET surface area). The amount measured may differ from the available surface area of the oxide in the solution [8]. This is not a disadvantage when using one type of oxide, but comparing the surface charges of different oxides with each other may be questionable.

After each addition of a portion of acid or base to the oxide dispersion, we must wait until the electrode readings stabilize. Depending on the criteria adopted, it may take minutes, hours, or even days. In laboratory practice, we usually shorten this period to several dozen minutes. This procedure, called fast titration, was used in our studies [11]. However, some researchers believe that it is necessary to wait much longer for the system to reach a state of true equilibrium [31], which, for example, may include a change in the crystalline phase of the oxide being tested. This second approach is called slow titration.

We determined the reversibility of hydrogen ion adsorption by examining titration curves expressed as the hydrogen surface charge as a function of pH. If the base and acid titration curves overlap, we can conclude that the adsorption of hydrogen ions is reversible. The reversibility of $H^{+}$ ion adsorption is influenced by the kinetics of this process. For example, in porous adsorbents, the transport of ions to the surface is slowed down, and hysteresis may appear in the titration curves.

When presenting experimental data, the values obtained during base titration are marked in blue and squares, while those obtained in reverse titration using acid are marked in red and circles. The solid concentration was equal to 10 g/dm^3^ while measuring the proton surface charge density and adsorption edges. During the electrokinetic measurements, the $TiO_{2}$ concentration was 1 g/dm^3^.

The data were collected in a pH range of 3 to 10, covering the area where the adsorption of copper ions begins and the adsorption of calcium ions ends. Titanium dioxide is practically insoluble in this pH range during measurements. The speciation analysis of $Ca^{2+}$ and $Cu^{2+}$ ions indicates that they remain in the solution as free ions until their adsorption.

**Figure 1:**
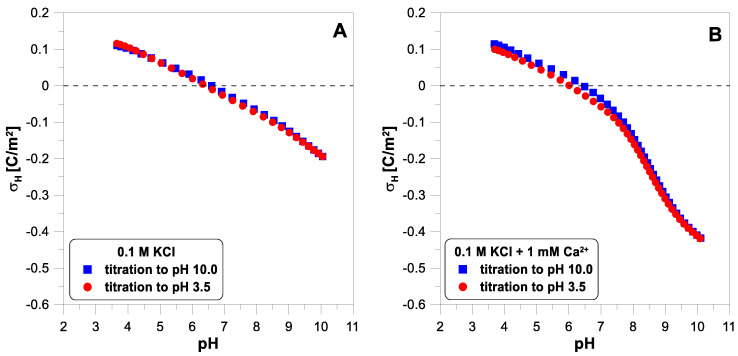
The proton surface charge density, σ_H_, of $TiO_{2}$ as a function of pH in 0.1 M of KCl electrolyte measured during forward and reverse titration: (**A**) without divalent ions; (**B**) with 1 mM of $CaCl_{2}$ .

Figure 1 presents the proton surface charge density, $\sigma_{H},$ of titanium dioxide as a function of pH measured in 0.1 M of KCl . Each titration run gives almost identical results. This confirms that hydrogen ion adsorption on titanium dioxide is a reversible process. Adding $Ca^{2+}$ ions to the system causes a significant reduction in the charge values at a pH above 7. Above this pH, there is a strong adsorption of calcium ions on the titanium dioxide surface (compare with the adsorption edge of calcium). In the presence of calcium ions, the reverse titration data points (red color—circles) lie a little below the forward titration points (blue color—squares) below a pH of 7.5. This may suggest that not all calcium ions were desorbed from the surface when the pH was decreased.

**Figure 2:**
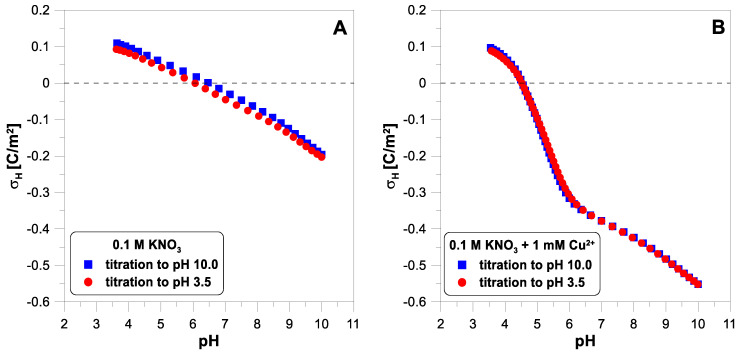
The proton surface charge density, σ_H_, of $TiO_{2}$ as a function of pH in 0.1 M of $KNO_{3}$ electrolyte measured during forward and reverse titration: (**A**) without copper ions; (**B**) with 1 mM of $Cu{\left(NO_{3}\right)}_{2}$ .

Figure 2 shows the net proton surface charge density measured in 0.1 M of $KNO_{3}$ electrolyte with copper ions (1 mM). The reversibility test of hydrogen ion adsorption reveals slight differences between the experimental points from both curves, especially in the middle of the pH range (Figure 2A). The addition of copper ions (Figure 2B) leads to a dramatic drop in the proton surface charge in the pH range from 4.5 to 6.5. Despite a very visible change in the course of the charge curve, we observe excellent reversibility of hydrogen ion adsorption. In Figure 2B, both sets of experimental points coincide almost perfectly.

**Figure 3:**
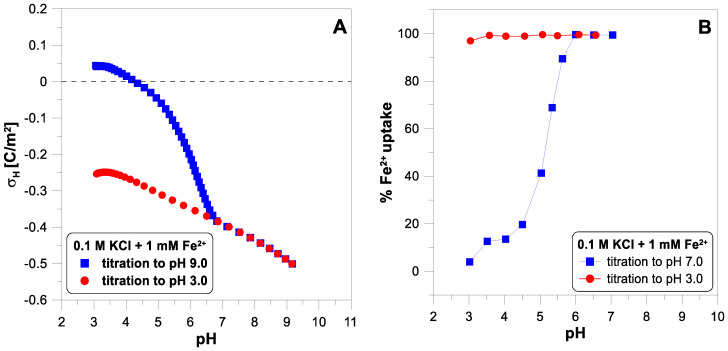
(**A**) The proton surface charge density, σ_H_, of $TiO_{2}$ as a function of pH in 0.1 M of KCl electrolyte with 1 mM of $FeCl_{2}$ measured during forward and reverse titration. (**B**) The adsorption edges of $Fe^{2+}$ ions.

Figure 3 presents the surface charge of $TiO_{2}$ in the presence of 1 mM of $Fe^{2+}$ and the adsorption edge for these ions. Below a pH of 6.5, we observe a vast difference between the two titration runs. Above this pH value, the experimental points overlap. This means that some irreversible process occurs when the pH increases. The Fe(II) adsorbs on the oxide surface, but above a pH of 5, it begins to oxidize very quickly to Fe(III), which undergoes hydrolysis [28]. Oxidized and hydrolyzed iron(III) cannot return to its original form during reverse titration.

**Figure 4:**
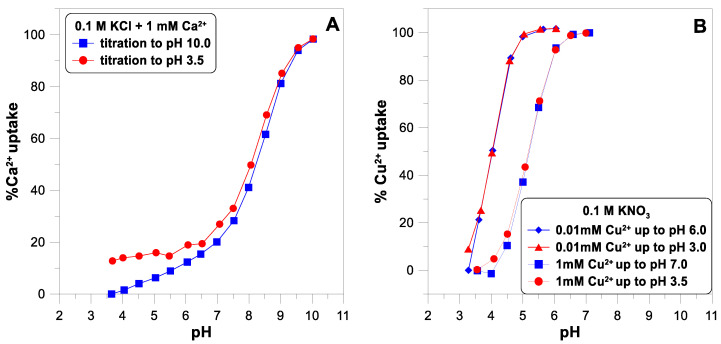
The adsorption edges of calcium (**A**) and copper (**B**) ions on titanium dioxide as a function of pH in 0.1 M of electrolyte measured during forward and reverse titration.

Figure 4 shows the adsorption edges for calcium and copper ions. The concentration of both ions was measured using the ion-selective electrodes. An ISE is a convenient and fast sensor for measuring the concentration of metal ions in an oxide suspension, giving results comparable to the spectrophotometric technique [11]. The desorption of calcium ions is not complete as the pH decreases (Figure 4A). The adsorption edge of the copper ions was measured for two different concentrations of $Cu^{2+}$ (Figure 4B). Some authors [10] suggest that a small part of the SOH groups on the oxide surface have a very high affinity for divalent ions and, at their very low concentrations, can bind them practically irreversibly. Figure 4B does not show any such manifestations of surface heterogeneity. In the case of a lower concentration of copper ions (0.01 mM), the adsorption edge is shifted towards lower pH values. For the higher concentration (1 mM), the edge starts at a pH of 4 and ends at a pH of 7. This is a much narrower range than the edge measured for the calcium ions (from pH 4 to pH 10).

Electrokinetic measurements, in which the electrophoretic mobility of charged particles moving in an electric field is determined, are an essential research technique used in colloid chemistry [33]. From the electrophoretic mobility data, using an appropriate theoretical model, we can calculate the electric potential established at the boundary of the moving particle and the stationary solution—the electrokinetic potential or ζ -potential. There are two classical limiting approximations for calculating this potential, as proposed by Smoluchowski and Huckel [30]. The first is used for large particles (with a diameter of several hundred nm) moving in a solution with a high ionic strength, and the second is for small particles (with a diameter of a few nm) moving in a solution with a low ionic strength.

Both approximations assume a linear relationship between the zeta potential and electrokinetic mobility. The zeta potential calculated from Huckel’s formula is simply 50% greater than that calculated from Smoluchowski’s approximation. A general and accurate theory describing the electrophoretic mobility of spherical colloidal particles was developed by O’Brien and White in 1978 [34].

Modern devices use light scattering to measure electrophoretic mobility. Combining laser Doppler velocimetry and phase analysis light scattering, this technique ensures accurate results with millions of particles observed. The disadvantage of this method is that the light scattering increases significantly with particle size, which leads to an overestimation of the contributions made by the largest particles in the case of polydisperse samples.

The magnitude of the zeta potential of oxide particles depends on the pH of the solution, the concentration of the indifferent electrolyte, and the multivalent ions adsorbed on their surface. Therefore, tracking the changes in this potential enables an indirect examination of the reversibility of the adsorption of multivalent ions.

**Figure 5:**
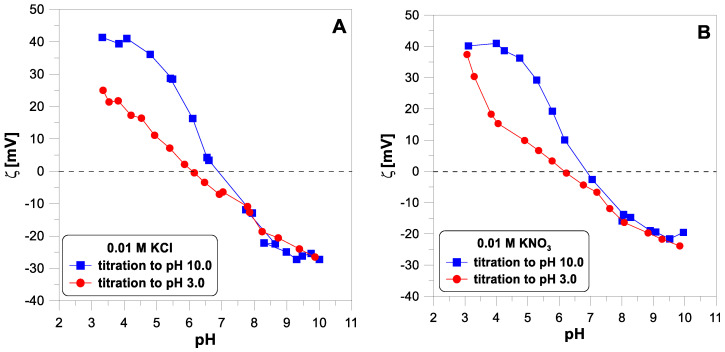
The ζ-potential of $TiO_{2}$ particles measured during forward and reverse titration for two different electrolytes: (**A**) 0.01 M of KCl and (**B**) 0.01 M of $KNO_{3}$ without divalent ions.

**Figure 6:**
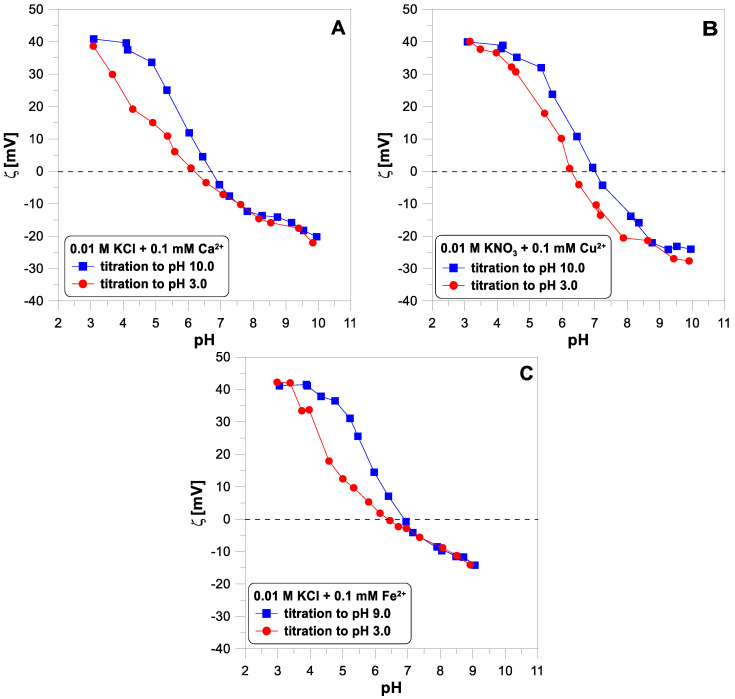
The ζ-potential of $TiO_{2}$ particles measured during forward and reverse titration in 0.01 M of electrolytes in the presence of divalent ions at a concentration of 0.1 mM: (**A**) $Ca^{2+}$ ; (**B**) $Cu^{2+}$ ; (**C**) $Fe^{2+}$ .

Figure 5 shows the electrokinetic potential measured for titanium dioxide particles with a concentration of 1 g/dm^3^ in the two electrolyte solutions, KCl and $KNO_{3}$ , with a concentration of 0.01 M without the addition of divalent ions. The points obtained from the base and acid titration differ for pH values below 7. Figure 6 shows the influence of divalent ions on the behavior of the zeta potential when changing the titration direction. In each of the three cases, we observe apparent hysteresis. The initial and final values coincide, but the experimental points diverge in the middle of the pH range. In each case, the zeta potential measured during the acid titration has lower values than during the base titration, below a pH of 7.

## 3. Discussion

The proton surface charge results presented above confirm that hydrogen ion adsorption is reversible with pH changes. On the other hand, the data divergence for the electrokinetic potential (Figure 5) when changing the direction of titration suggests non-reversibility. According to Lyklema [7]: “Surface charges and electrokinetic charges are very different double layer characteristics. It is mandatory to discriminate between them. If for a given system both of them are known, much relevant double layer information becomes accessible”.

To explain the lack of reversibility of the electrokinetic curves with a change in the titration direction, it should be noted that electrophoretic mobility is a dynamic quantity that may depend on the instantaneous size of the moving particles. Titanium dioxide nanoparticles may agglomerate in a solution as the pH changes. Especially in the pH region close to the IEP (isoelectric point), where the charge of the particles is close to zero, they can easily combine into larger structures. The resulting agglomerates can then disintegrate as the pH changes further and the surface charge increases.

We performed $TiO_{2}$ particle size measurements with the Zetasizer Nano instrument for three pH values (beginning of titration, near IEP, end of titration). The particle size (Z-average) measured at a pH of 3.0, 6.6, and 9.1 was 824 nm, 4112 nm, and 2140 nm, respectively. The particle size increases significantly near the IEP. The particle size in the solution is two orders of magnitude larger than that reported by the manufacturer. According to the $TiO_{2}$ powder manufacturer, the average particle size is 21 nm. Such a significant difference may result from the use of measurement techniques. Light scattering techniques provide information about the hydrodynamic diameter of aggregates in a solution, whereas a TEM image analysis gives the diameter of the primary particle. Additionally, the incident light is scattered particularly strongly by the largest particles in the solution, which means that the diameter determined by this method is similar to the size of the largest aggregates.

The process described above has little effect on the adsorption values of hydrogen ions ( $\sigma_{H}$ ) and divalent ions because these are equilibrium values (or quasi-equilibrium). However, this phenomenon may slightly delay the establishment of equilibrium in a solution.

It was found that the zeta potential of oxide nanoparticles depends on their concentration [35]. At a higher concentration, the ζ(pH) curve shifts towards higher pH values. The authors of that publication concluded that too low an oxide concentration makes its particles sensitive to impurities like bicarbonate ions in the solution.

Another possible explanation for the observed discrepancies may be the increase in ionic strength during the reversible titration. The salt formed during titration increases the electrolyte concentration. Increasing the ionic strength causes a decrease in the measured electrokinetic potential.

Since the lack of reversibility of the electrokinetic potential is observed in a system containing no divalent ions, the ζ potential behavior cannot be a reliable test for the reversibility of the adsorption of these ions. Looking at Figure 6, it is difficult to say which of the divalent ions adsorb irreversibly.

A more reliable method for assessing adsorption reversibility is measuring the proton surface charge density, which depends on pH and the amount of adsorbed divalent ions. Namely, the adsorbed cations displace protons from SOH groups and undergo hydrolysis at the surface [11,27], increasing the amount of hydrogen ions released into the bulk solution (Equation (1)).

(1) $$SOH+M^{2+}+H_{2}O\overset{\text{​}}{\to }SOM\left(OH\right)+2H^{+}$$

According to Reaction (1), this results in the release of a large number of hydrogen ions from the surface to the solution and a significant reduction in its hydrogen charge, $\sigma_{H}$ . Generally speaking, the data in Figure 1B and Figure 2B do not represent the net total charge on the surface, $\sigma_{0}$ , only the charge generated by hydrogen ions, $\sigma_{H}$ . However, both charges can be equal when no specific adsorption occurs.

As we mentioned, we carried out a so-called fast titration, where the waiting time between subsequent additions of a base or acid was up to several dozen minutes. The double-layer relaxation time for the hematite/water interface determined by electrochemical impedance spectroscopy was about a few seconds [36]. Therefore, the time intervals applied in the fast titration appear to achieve equilibrium at the oxide/electrolyte interface.

The drop in surface charge in the presence of copper ions is steeper and occurs at a lower pH than in the case of calcium ions. The probable cause of this phenomenon is the stronger hydrolysis of $Cu^{2+}$ than of $Ca^{2+}$ on the oxide surface.

Figure 3 shows the course of the surface charge and adsorption edge in a system where an irreversible redox reaction (Equation (2)) occurs [28]. For this reason, the system is not pH-reversible. It should be emphasized that it is impossible to determine whether some irreversible process occurs with a change in pH by analyzing only a one-way titration using an acid or a base. It is also worth noting that if we started the titration of this system at a pH of 9 to a pH of 3, we would probably find the system reversible.

(2) $${Fe}^{2+}+{3H}_{2}O\overset{\text{​}}{\to }Fe{\left(OH\right)}_{3}+3H^{+}+1e^{-}$$

It is worth emphasizing that electrically charged oxide nanoparticles (like $TiO_{2}$ NPs) show extraordinary ionic reactivity [37]. The surface charge density of titanium dioxide nanoparticles is much higher than for larger particles [32]. Therefore, particle size should be considered when calculating the electrokinetic potential from mobilities.

In summary, it can be said that proton surface charge density measurements in forward and reverse titration are a simple and reliable test for the reversibility of divalent ion adsorption. However, when the amount of these ions is too low, only a direct measurement of their concentration along with pH changes allows us to assess the reversibility of their adsorption. The electrokinetic potential of oxide particles is very sensitive to the adsorption of multivalent ions. Still, the complex and unpredictable effect of pH on the electrophoretic mobility of nanoparticles does not allow this technique to determine the irreversibility of ion adsorption.

## 4. Materials and Methods

Titanium dioxide, which we used in our experiments, was provided by Evonik, Essen, Germany. It was Aeroxide P25, commercial hydrophilic fumed $TiO_{2},$ consisting of anatase and rutile with a weight ratio of 80:20. According to the manufacturer, its specific surface area is 50 ± 15 m^2^·g^−1^ (BET), and the average size of particles is about 21 nm. In our experiments, we used unmodified $TiO_{2}$ nanopowder without additional purification. The titanium dioxide may have had some acidic impurities ( HCl ) coming from its synthesis process (the hydrolysis of $TiCl_{4}$ ). Possible traces of HCl , presented in $TiO_{2}$ , can be eliminated during data processing [9]. The same procedure was applied in our earlier paper [11].

Inert electrolyte solutions ( KCl and $KNO_{3}$ ) were prepared with analytical-grade salts. We chose potassium salts because K^+^ ions do not interfere with the glass electrode or the calcium ion-selective electrode. The acid and base solution used in the titration (0.1 M of HCl , 0.1 M of $HNO_{3},$ and 0.1 M of KOH ) was prepared from the analytical weights of the reagents by dilution with water in 1000 mL flasks. The stock solutions of $CaCl_{2}$ , $Cu{\left(NO_{3}\right)}_{2}\cdot 3H_{2}O$ , $FeCl_{2}\cdot 4H_{2}O$ were freshly prepared before measurements. Milli-Q quality water, which was decarbonized and deoxygenated by passing pure argon, was used to prepare the above-mentioned solutions.

All potentiometric titrations were conducted using the Titrando 907 instrument (Metrohm, Herisau, Switzerland) with two measuring interfaces and two 800 Dosino dosing units (containing an acid and a base). The whole titration course was controlled by the Metrohm Tiamo 2.5 software. The glass-jacketed stirring vessel, where titration took place, was fitted with a tube to purge the solution with pure argon (to remove $CO_{2}$ traces). A Julabo F12 thermostat was applied to keep the temperature of the solution at 25 °C. The pH was measured with the combined glass electrode (Unitrode from Metrohm). The fixed drift limit (0.5 mV·min^−1^) was used to save the final pH value in each titration step. The glass electrode was calibrated at pH = 9, pH = 7, and pH = 4, with three buffer solutions (from Metrohm). The exact titer of the applied acid and base solutions was also determined.

We placed in the titration vessel 100 cm^3^ of 0.1 M of inert electrolyte solution; next, we added 1 g of $TiO_{2}$ to obtain an oxide concentration of 10 g·dm^−3^; and finally, we added the calculated amount of the stock solution containing divalent metal ions to obtain a concentration of 1 mM of $Me^{2+}$ . In the first step of each titration, 0.5 cm^3^ of 0.1 M of acid was added to a 100 cm^3^ suspension. Then, the system was equilibrated to reach a pH of about 3–4 and next titrated with KOH to pH 10 (the forward titration). Finally, the reverse titration was performed with acid ( HCl or $HNO_{3}$ ) with the same parameters as the forward titration. A detailed discussion of the titration of a metal oxide suspension can be found in the literature [8].

We determined the net proton balance in the studied system to obtain the proton surface charge density, σ_H_, [28]:

(3) $$\sigma_{H}\left[\frac{C}{m^{2}}\right]=\frac{F}{A_{s}C_{s}}\left[\left(\frac{C_{A}{\cdot ∆V}_{A}}{V_{susp}}-\frac{1}{\gamma_{H^{+}}}10^{-pH}\right)-\left(\frac{C_{B}\cdot {∆V}_{B}}{V_{susp}}-\frac{1}{\gamma_{{OH}^{-}}}10^{pH-14}\right)\right]$$

where F is the Faraday constant [C·mol^−1^], $A_{s}$ is the specific surface area [m^2^·g^−1^], $C_{s}$ is the oxide concentration in the suspension [g·dm^−3^], $C_{A}\;and\;C_{B}$ are the concentrations of the HCl (or $HNO_{3}$ ) and KOH solutions [M], ${∆V}_{A}\;and\;{∆V}_{B}$ are the added volumes of HCl (or $HNO_{3}$ ) and KOH [dm^3^], $\gamma_{H^{+}}\;and\;\gamma_{{OH}^{-}}$ are the activity coefficients of the $H^{+}$ and $OH^{-}$ ions, and $V_{susp}$ is the oxide suspension volume [dm^3^].

The calcium ion concentration was measured by the direct potentiometry method [29] using a Ca-ISE with a polymer membrane (Metrohm No. 6.0508.110) and a reference electrode (LL ISE Reference, Metrohm No. 6.0750.100). The measuring range of the Ca-ISE is from 5·10^−6^ M to 1 M, and the pH range is from 2 to 12.

The copper ion concentration was measured using a Cu-ISE with a crystal membrane (Metrohm No. 6.0502.140) and a reference electrode, the same as for the Ca-ISE. The measuring range of the Cu-ISE is from 1·10^−8^ M to 0.1 M, and the pH range from 2 to 12.

Both ion-selective electrodes were calibrated using four standards for $Ca^{2+}$ and $Cu^{2+}$ ions (10, 1, 0.1, 0.01 mM) dissolved in a 0.1 M KCl or 0.1 $KNO_{3}$ solution. The drift limit for the electrodes applied in this part of the experiment was established as below 0.2 mV·min^−1^.

In the adsorption experiment with $Fe^{2+}$ , we used the spectrophotometric method to quantify the amount of iron remaining in the solution. Small aliquots of the suspension containing iron ions were collected once the system reached equilibrium (i.e., after the stabilization of the pH and redox potential) and centrifuged to remove the solid phase. Next, we used a spectrophotometer (Evolution 201 from ThermoFisher Scientific, Waltham, MA, USA), applying the method with o-phenanthroline to determine the $Fe^{2+}$ ion concentration [28]. All titration and adsorption experiments were conducted at a temperature of 25 °C.

In the electrokinetic measurements, we applied the Zetasizer Nano ZS (Malvern Pananalytical, Malvern, UK) combined with the MPT-2 autotitrator to measure the electrokinetic potential (ζ) of the titanium dioxide suspension as a function of pH. The particle velocity is determined by measuring the phase shift, known as phase analysis light scattering (PALS). The light scattered is combined with the reference beam. This produces a fluctuating intensity signal where the fluctuation rate is proportional to the speed of the particles. After determining the electrophoretic mobility, the ζ-potential is then calculated using the Smoluchowski equation [30]:

(4) $$\zeta =\frac{\eta \mu }{\varepsilon }$$

where ζ is the electrokinetic potential, η is the viscosity, μ is the electrophoretic mobility, and ε is the dielectric constant of a solution.

More dilute samples than in the adsorption experiments should be analyzed to obtain stable data from the apparatus. Electrophoretic measurements at a high particle concentration yield unreliable results [35]. Therefore, the concentration of $TiO_{2}$ suspension was 1 g·dm^−3^ in 0.01 M of inert electrolytes with 0.1 mM of divalent metal ions [11]. The above values are ten times lower than the parameters used during the adsorption experiments.

The uncertainty in the pH measurements during titration is at least ± 0.02 pH unit, which gives an uncertainty in the hydrogen ion concentration of 5% and an error in the charge measurement of 0.005–0.01 $C/m^{2}$ . The error in measuring the concentration of divalent ions is also at the level of 5%. The uncertainty in the electrokinetic measurements is approximately 10%.

## Data Availability

Data are contained within the article.

## References

[1] Morel F.M.M., Hering J.G. (1993). Principles and Applications of Aquatic Chemistry.

[2] Stumm W., Morgan J.J. (1996). Aquatic Chemistry: Chemical Equilibria and Rates in Natural Waters.

[3] Yates D.E., Levine S., Healy T.W. (1974). Site-binding model of the electrical double layer at the oxide/water interface. J. Chem. Soc. Faraday Trans..

[4] Davis J.A., James R.O., Leckie J.O. (1978). Surface ionization and complexation at the oxide/water interface I. Computation of electrical double layer properties in simple electrolytes. J. Colloid Interface Sci..

[5] Davis J.A., Kent D.B., Hochella M.F., White A.F. (1990). Surface complexation modeling in aqueous geochemistry. Mineral-Water Interface Geochemistry.

[6] Raji Z., Karim A., Karam A., Khalloufi S. (2023). Adsorption of Heavy Metals: Mechanisms, Kinetics, and Applications of Various Adsorbents in Wastewater Remediation—A Review. Waste.

[7] Lyklema J. (2011). Surface charges and electrokinetic charges: Distinctions and juxtapositionings. Colloids Surf. A Physicochem. Eng. Asp..

[8] Lützenkirchen J., Preočanin T., Kovačević D., Tomišić V., Lövgren L., Kallay N. (2012). Potentiometric Titrations as a Tool for Surface Charge Determination. Croat. Chem. Acta.

[9] Szekeres M., Tombacz E. (2012). Surface charge characterization of metal oxides by potentiometric acid-base titration, revisited theory and experiment. Colloids Surf. A Physicochem. Eng. Asp..

[10] Dzombak D.A., Morel F.M.M. (1990). Surface Complexation Modeling.

[11] Szymanek K., Charmas R., Piasecki W. (2020). A study on the mechanism of Ca^2+^ adsorption on TiO_2_ and Fe_2_O_3_ with the usage of calcium ion-selective electrode. Chemosphere.

[12] Schultz M.F., Benjamin M.M., Ferguson J.F. (1987). Adsorption and Desorption of Metals on Ferrihydrite: Reversibility of the Reaction and Sorption Properties of the Regenerated Solid. Environ. Sci. Technol..

[13] Perez F.F., Sweeck L., Bauwens W., Hees M.V., Elskens M. (2015). Adsorption and desorption kinetics of Co-60 and Cs-137 in fresh water rivers. J. Environ. Radioact..

[14] Turner L.J., Kramer J.R. (1992). Irreversibility of Sulfate Sorption on Goethite and Hematite. Water Air Soil Pollut..

[15] Backes C.A., McLaren R.G., Rate A.W., Swift R.S. (1995). Kinetics of cadmium and cobalt desorption from iron and manganese oxides. Soil Sci. Soc. Am. J..

[16] Machesky M.L., Jacobs P.F. (1991). Titration calorimetry of aqueous alumina suspensions Part I. Results and comparison with similar studies. Colloids Surf..

[17] Preocanin T., Cop A., Kallay N. (2006). Surface potential of hematite in aqueous electrolyte solution: Hysteresis and equilibration at the interface. J. Colloid Interface Sci..

[18] Piasecki W. (2003). Theoretical Description of the Kinetics of Proton Adsorption at the Oxide/Electrolyte Interface Based on the Statistical Rate Theory of Interfacial Transport and the 1pK Model of Surface Charging. Langmuir.

[19] Kosmulski M. (2001). Chemical Properties of Material Surfaces.

[20] Ezati F., Sepehr E., Ahmadi F. (2021). The efficiency of nano-TiO_2_ and γ-Al_2_O_3_ in copper removal from aqueous solution by characterization and adsorption study. Sci. Rep..

[21] Kang X., Liu S., Dai Z., He Y., Song X., Tan Z. (2019). Titanium Dioxide: From Engineering to Applications. Catalysts.

[22] Kefi B.B., Bouchmila I., Martin P., M’Hamdi N. (2022). Titanium Dioxide Nanotubes as Solid-Phase Extraction Adsorbent for the Determination of Copper in Natural Water Samples. Materials.

[23] Khomami N.T.S., Welle A., Kunz S., Philippe A. (2022). Sorption of Fulvic Acids onto Titanium Dioxide Nanoparticles Extracted from Commercial Sunscreens: ToF-SIMS and High-Dimensional Data Analysis. Coatings.

[24] Racovita A.D. (2022). Titanium Dioxide: Structure, Impact, and Toxicity. Int. J. Environ. Res. Public Health.

[25] Alhalili Z. (2023). Metal Oxides Nanoparticles: General Structural Description, Chemical, Physical, and Biological Synthesis Methods, Role in Pesticides and Heavy Metal Removal through Wastewater Treatment. Molecules.

[26] Khalameida S., Skwarek E., Janusz W., Sydorchuk V., Leboda R., Skubiszewska-Zięba J. (2014). Electokinetic and adsorption properties of different titanium dioxides at the solid/solution interface. Cent. Eur. J. Chem..

[27] Sverjensky D.A. (2006). Prediction of the speciation of alkaline earths adsorbed on mineral surfaces in salt solutions. Geochim. Cosmochim. Acta.

[28] Piasecki W., Szymanek K., Charmas R. (2019). Fe^2+^ adsorption on iron oxide: The importance of the redox potential of the adsorption system. Adsorption.

[29] Lindner E., Pendley B.D. (2013). A tutorial on the application of ion-selective electrode potentiometry: An analytical method with unique qualities, unexplored opportunities and potential pitfalls; Tutorial. Anal. Chim. Acta.

[30] Doane T.L., Chuang C.-H., Hill R.J., Burda C. (2012). Nanoparticle ζ-Potentials. Acc. Chem. Res..

[31] Duc M., Adekola F., Lefèvre G., Fédoroff M. (2006). Influence of kinetics on the determination of the surface reactivity of oxide suspensions by acid–base titration. J. Colloid Interface Sci..

[32] Holmberg J.P., Ahlberg E., Bergenholtz J., Hassellov M., Abbas Z. (2013). Surface charge and interfacial potential of titanium dioxide nanoparticles: Experimental and theoretical investigations. J. Colloid Interface Sci..

[33] Hunter R.J. (1989). Foundations of Colloid Science.

[34] O’Brien R.W., White L.R. (1978). Electrophoretic mobility of a spherical colloidal particle. J. Chem. Soc. Faraday Trans. 2.

[35] Wang N., Hsu C., Zhu L., Tseng S., Hsu J.P. (2013). Influence of metal oxide nanoparticles concentration on their zeta potential. J. Colloid Interface Sci..

[36] Shimizu K., Boily J.F. (2014). Electrochemical Properties and Relaxation Times of the Hematite/Water Interface. Langmuir.

[37] van Leeuwen H.P., Buffle J., Duval J.F.L., Town R.M. (2013). Understanding the Extraordinary Ionic Reacitivity of Aqueous Nanoparticles. Langmuir.

